# *Ex Vivo*^1^H NMR study of pituitary adenomas to differentiate various immunohistochemical subtypes

**DOI:** 10.1038/s41598-019-38542-6

**Published:** 2019-02-28

**Authors:** Omkar B. Ijare, David S. Baskin, Kumar Pichumani

**Affiliations:** 1 Kenneth R. Peak Brain and Pituitary Tumor Treatment Center, Department of Neurosurgery, Houston Methodist Neurological Institute, Houston Methodist Hospital and Research Institute, Houston, TX USA; 2 Weill Cornell Medical College, New York, NY USA

## Abstract

Pituitary adenomas (PAs) are benign growths arising from epithelial cells in the adenohypophysis of the pituitary gland. To date, there has been no detailed metabolic characterization of PAs of various subtypes. In this study, we report nuclear magnetic resonance (NMR) based metabolomic analysis of surgically resected tumors from forty five pituitary tumor patients [gonadotropic (LH/FSH-secreting) = 17; prolactinomas (PRL-secreting) = 11, Cushing’s disease (ACTH-secreting) = 4, non-functional = 5, and mixed = 8] who underwent transsphenoidal selective adenomectomy. Compared to LH/FSH-secreting tumors, PRL-secreting tumors showed statistically significant decrease in the levels of N-acetylaspartate (NAA), myo-inositol (mI), scyllo-inositol (sI), glycine, taurine, phosphoethanolamine (PE) and increase in the levels of glutamine. When compared with LH/FSH-secreting tumors, ACTH-secreting tumors showed statistically significant decrease in the levels of sI, glycine, PE and increase in the levels of aspartate. Although lipid extracts of PAs showed the presence of many common lipid molecules, only glycerophosphoethanolamine (GPE) showed statistically significant decrease in PRL, ACTH and non-functional subtypes when compared to LH/FSH-secreting tumors. Changes observed in these metabolite concentrations among various subtypes of PAs reflect metabolic heterogeneity in these tumors and may pave the way towards the development of metabolic markers to distinguish various immunohistochemical subtypes of PAs.

## Introduction

The pituitary gland secretes critical hormones including growth (GH), adrenocorticotropic (ACTH), prolactin (PRL), luteinizing (LH), follicle-stimulating (FSH), and thyroid-stimulating (TSH) which regulate the majority of the endocrine system^1^. Pituitary adenomas (PAs) are considered to be the benign growth of cells of the anterior lobe (adenohypophysis) of the pituitary gland^2^. Some PAs may be more aggressive and grow rapidly in size (macroadenomas) while others may metastasize intracranially and are considered as pituitary carcinomas^2–4^. PAs arising from adenohypophyseal cells are clinically classified as either functional (tumors showing immunoreactivity for the enzymes LH, FSH, PRL, ACTH, TSH and GH) or non-functional tumors that do not secrete active hormones^4,5^. PAs may interfere with normal production of one or more of the pituitary hormones as they grow and compress and/or destroy the normal pituitary gland. The growth of PAs and hypersecretion of hormones can produce a number of clinical symptoms, the consequences of which produce clinical syndromes and therapeutic challenges^3,4^. Although routine diagnostic magnetic resonance imaging (MRI) is used to diagnose most PAs, it may not detect microadenomas unambiguously. The lack of suitable experimental model systems such as patient-derived cell lines and animal models limit our current understanding of pituitary tumorigenesis, which further impedes the development of new molecular therapeutic targets and biomarkers for diagnostic and prognostic purposes.

Metabolomics provides a terminal view of biological processes such as gene transcription, post translational modifications, enzymatic expressions, and activation. Small changes in enzyme activity directly lead to changes in metabolic fluxes, which in turn produces larger changes in metabolite concentrations. Therefore, metabolomic studies of pituitary tumors may provide new biological insights concerning various subtypes of pituitary tumors, and identify novel therapeutic targets and small molecule biomarkers.

Nuclear magnetic resonance (NMR) spectroscopy and mass spectrometry (MS) are the two major analytical platforms that are commonly used for metabolomics analysis of biofluids and tissue specimens^6–8^. Although both techniques are complementary to each other, NMR spectroscopy has a unique advantage of being non-destructive including its capability to translate *ex vivo* observations into *in vivo* applications using clinical MRI scanners in noninvasive diagnostics. Although NMR spectroscopy has been widely used for the characterization of brain tumors^3,9,10,11^, there has not been a detailed metabolomic investigation of various immunohistochemical subtypes of pituitary tumors. In the present study, we have characterized both aqueous and lipid metabolites that are present in PAs using *ex vivo* ^1^H NMR spectroscopy. Further, we have identified a set of metabolites that can be used to distinguish PAs of various immunohistochemical subtypes.

## Materials and Methods

### Patients

Forty five pituitary adenoma tissue samples from various immunohistochemical subtypes (Table 1) were collected from patients undergoing transsphenoidal selective adenomectomy at the Houston Methodist Hospital. All surgeries were performed by the neuro-surgeon and the co-author (DSB). Informed consent was obtained from each patient following an Institutional Review Board protocol approved by Houston Methodist Hospital and Research Institute. All methods were carried out in accordance with the relevant guidelines and regulations. The clinical and histopathological characteristics of study participants are provided in Table 1. All patients that are included in this study showed a low MIB-1 index (indicative marker of cellular proliferation) of less than 6%.

**Table 1:** Patients characteristics showing tumor type, and MIB-1 index.

Patient	Sex	Age (years)	Tumor type (Immunohistochemistry)	MIB-1 (%)
1	M	45	LH	2
2	M	82	FSH	3
3	F	67	FSH	1
4	F	64	FSH	1
5	M	62	FSH	1
6	M	46	FSH	2-3
7	M	66	LH/FSH	2
8	M	68	LH/FSH	1
9	M	62	LH/FSH	2
10	F	63	LH/FSH	2
11	F	66	LH/FSH	3
12	F	25	LH/FSH	2
13	F	54	LH/FSH	1
14	M	53	LH/FSH	2
15	M	61	LH/FSH	1-2
16	M	47	LH/FSH	1
17	F	65	LH/FSH	1
18	M	66	PRL	1
19	F	26	PRL	1
20	M	51	PRL	1
21	F	49	PRL	3-4
22	F	34	PRL	2
23	F	18	PRL	2-3
24	F	36	PRL	3
25	F	22	PRL	NA
26	F	50	PRL	1
27	F	37	PRL	1-2
28	F	45	PRL	2
29	F	37	ACTH	1
30	M	70	ACTH	NS
31	F	32	ACTH	2-3
32	F	54	ACTH	2-4
33	M	61	Mixed (FSH, PRL)	1
34	F	49	Mixed (FSH, PRL, TSH)	1
35	F	62	Mixed (PRL, GH)	2
36	F	68	Mixed (GH, LH, FSH, PRL)	1
37	F	49	Mixed (ACTH, LH, FSH)	3
38	F	48	Mixed (GH, PRL)	2-6
39	M	73	Mixed (GH, ACTH, PRL)	1
40	M	45	Mixed (ACTH, LH, FSH)	1-2
41	F	46	Negative	1
42	M	37	Negative	3
43	F	39	Negative	1
44	M	42	Negative	1
45	F	75	Negative	1

Negative, negative for all of the hormones secreted by pituitary adenomas; NA, not available; NS, no staining.

#### Chemicals

Methanol, chloroform, and chloroform-*d* (CDCl_3_) were purchased from Millipore Sigma (St. Louis, MO, USA). D_2_O, DCl, and NaOD were purchased from Cambridge isotope laboratories (Tewksbury, MA, USA). 3-(trimethylsilyl)-1-propane sulfonic acid-d_6_ sodium salt (DSS-d_6_) solution was purchased from Chenomx Inc. (Edmonton, Canada).

### Methanol-Chloroform extraction

Previous NMR studies on the analysis of brain tumors have confirmed that methanol:chloroform extraction method is advantageous than perchloric acid extraction^12^. In this study,we used similar methodology for the extraction of pituitary adenomas. Pre-weighed pituitary tumor tissue samples (~20 mg) were taken in extraction tubes containing zirconium oxide (ZrO_2_) beads (Benchmark Scientific, Edison, NJ, USA), and 0.5 mL of methanol:chloroform solvent mixture in the ratio 2:1 (kept at 4 °C) was added to the tube containing the tumor tissue. The tissue sample was left in contact with the solvent mixture for 5 minutes (kept on ice) and then the tubes were spun at 4000 rpm for 2 minutes using a tissue homogenizer, Bead Bug (Benchmark Scientific, Edison, NJ, USA). To avoid any decomposition of tissue metabolites (due to heat generated during homogenization), the tubes containing tumor extracts were kept on ice for 2 minutes. The above step was repeated 3–4 times or until the tumor tissue is totally broken down to give a homogenate. Later, 0.25 mL of chloroform and 0.25 mL of Millipore water (1:1 ratio, v/v, 4 °C) were added to the tissue extract and the tubes were spun for an additional 2 minutes. Finally, the aqueous-methanol and chloroform layers were separated by centrifugation (RCF = 10,000 × g, 10 minutes) to obtain aqueous and lipid components respectively. The solvents were dried in a CentriVap^®^ vacuum concentrator (Labconco corporation, Kansas City, MO) and the residue from methanol layer was reconstituted in 180 µL D_2_O containing 1.0 mM DSS-d_6_ (internal standard), and the pH of the solution was adjusted to 7.4 ± 0.05. Residue from chloroform layer was redissolved in 180 µL CDCl_3_ containing 1% tetramethylsilane (TMS) (internal standard).

### 1D and 2D ^1^H NMR experiments

One-dimensional (1D) ^1^H and two-dimensional (2D) ^1^H-^1^H total correlated spectroscopy (TOCSY) NMR spectra of pituitary tumor extracts were collected on a Bruker 600 MHz spectrometer (^1^H Larmor frequency) equipped with a cryogenically-cooled ^1^H/^13^C detection probe (Bruker Biospin, Billerica, MA). The tumor extracts were taken in a 3 mm NMR tube and the ^1^H NMR spectra were obtained by using a pulse sequence which makes use of nuclear Overhauser effect with pre-saturation of water signal during relaxation and mixing times for the aqueous samples.  In the case of lipid extracts, a single non-selective radiofrequency pulse with the flip angle of 30º was used. The following acquisition parameters were employed: number of scans = 128, 90◦ pulse = 10 µs, number of points in time domain = 32 k, inter-pulse delay = 2.0 s, spectral width = 9,615 Hz, acquisition time = 1.70 s, mixing time = 100 ms and line broadening for exponential window function = 0.3 Hz. For ^1^H spectra, DSS-d_6_ (0 ppm) was used as an internal chemical shift and concentration reference. Peak areas of ^1^H signals were measured by using Bruker TopSpin 3.5 software. 2D ^1^H–^1^H TOCSY experiments were performed on tumor extracts to identify some of the overlapping proton signals from glutamate, glutamine, taurine, phosphocholine (PC), glycerophosphocholine (GPC) and phosphoethanolamine (PE) unambiguously. The parameters used were: spectral width = 9,590.8 Hz in both dimensions; time domain data points = 2,048; number of free induction decays with t_1_ increments = 400; relaxation delay = 2 s and number of transients = 24. A spin lock time of 80 ms was used for TOCSY experiments. Phase-sensitive data were obtained by the time proportional phase increment method. The resulting data were zero filled to 1,024 points in the t_1_-dimension and Fourier transformed along both the dimensions.

### Quantification of aqueous and lipid molecules in pituitary adenomas

We have identified aqueous metabolites (in the methanol-water layer) and lipid components (in the chloroform layer) by analyzing 1D ^1^H and 2D ^1^H-^1^H TOCSY NMR spectra in combination with comparison of chemical shifts reported in the literature^3,9–15^.

^1^H NMR spectral data from methanol and chloroform extracts of pituitary tumors were collected in the presence of internal standards DSS-d_6_ (for methanol-water extract) and TMS (chloroform extract). All ^1^H spectra were preprocessed and peak areas of metabolites and lipid components were measured and normalized to the peak area of respective internal standard. The concentrations of aqueous metabolites and lipid components were determined using the following equation^14^:

1 $${\rm{Analyte}}\,({\rm{mM}})=\frac{{\rm{mass}}\,{\rm{of}}\,{\rm{internal}}\,{\rm{standard}}\,({\rm{mg}})}{{\rm{MW}}\,{\rm{of}}\,{\rm{internal}}\,{\rm{standard}}}\ast \frac{\#\mathrm{protons}\,{\rm{in}}\,{\rm{internal}}\,{\rm{standard}}}{\#\mathrm{protons}\,{\rm{in}}\,{\rm{Analyte}}}\ast \frac{{\rm{Peak}}\,{\rm{area}}\,{\rm{of}}\,{\rm{the}}\,{\rm{Analyte}}}{{\rm{Peak}}\,{\rm{area}}\,{\rm{of}}\,{\rm{the}}\,{\rm{internal}}\,{\rm{standard}}}\ast \frac{1000}{{\rm{volume}}\,{\rm{of}}\,{\rm{sample}}\,{\rm{solution}}\,({\rm{mL}})}$$

Finally, metabolite concentrations were expressed in µmol/g of wet weight of the tumor tissue used in this analysis.

### Statistical Analysis

The means and standard deviations of all the quantified metabolites and the lipid components were compared between various PAs using Student’s t-test. Aqueous metabolites and lipids components showing statistical significance (p < 0.05) were identified.

## Results and Discussion

### Aqueous metabolites

**Figure 1:**
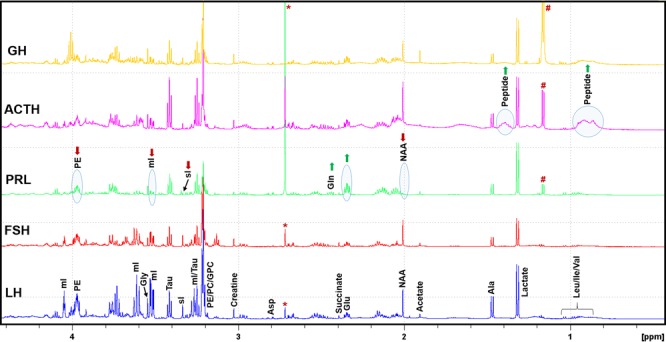
Typical ^1^H NMR spectra of aqueous layer of methanol:chloroform (2:1, v/v) extracts of LH, FSH, PRL, ACTH and GH secreting pituitary tumors showing relative levels of various metabolites. It should be noted that PRL secreting tumors showed decreased levels of NAA, sI, and mI compared to other types of tumors. ACTH secreting tumors are characterized by the elevated levels of peptide signals arising from elevated ACTH hormone in these tumors. LH, luteinizing hormone; FSH, follicle stimulating hormone; PRL, prolactin; ACTH, adrenocorticotropic hormone; GH, growth hormone; NAA, N-acetylaspartate; PE, phosphoethanolamine, sI, scyllo-inositol;, mI, myo-inositol; PC, phosphocholine; GPC, glycerophosphocholine; (*: solvent impurity; #: this peak is assigned to isopropanol).

Figure 1 shows representative ^1^H NMR spectra of various pituitary tumor subtypes. In the aqueous methanolic extract, we identified and quantified the following metabolites: branch chain amino acids (leucine, isoleucine, and valine), lactate, alanine, acetate, N-acetylaspartate (NAA), glutamate, succinate, glutamine, aspartate, creatine (Cr)/phosphocreatine (PCr), myo-inositol (mI), scyllo-inositol (sI), taurine, glycine, PE, and glucose. Signals from choline/PC/GPC (-N(CH_3_)_3_, 3.19–3.22 ppm) were overlapping with PE (-NCH_2_) signal at 3.21 ppm whereas betahydroxybutyrate (BHB) at 1.19 ppm was overlapping with an exogenous signal (assigned to isopropanol) at 1.16 ppm. As a result, these compounds were not included in the quantitative analysis.

^1^H NMR spectral profiles of LH and FSH secreting tumors looked similar whereas PRL showed decreased levels of NAA, mI, sI, and PE and elevated levels of glutamate and glutamine (Fig. 1). On the other hand, ACTH-secreting tumors showed decreased levels of sI, mI, glycine and PE when compared to LH and FSH. NAA and sI were increased in ACTH-secreting tumors when compared with PRL. Appearance of broad peaks at 0.75 ppm and 1.40 ppm was observed only in ACTH- secreting tumors. These tumors also showed the elevated levels of phenylalanine and tyrosine residues (broad proton signals at 6.70–7.40 ppm, Supplementary Figure S1). All these broad signals could be arising from the elevated levels of ACTH peptide in PAs in Cushing disease patients. These Cushing disease-specific ^1^H NMR signatures can be used to differentiate Cushing disease from other functional or non-functional PAs. In particular, the signals from phenylalanine and tyrosine residues appearing in the region 6.70–7.40 ppm of ^1^H NMR spectrum could be used to quantify the levels of ACTH hormone in pituitary tumors.

**Figure 2:**
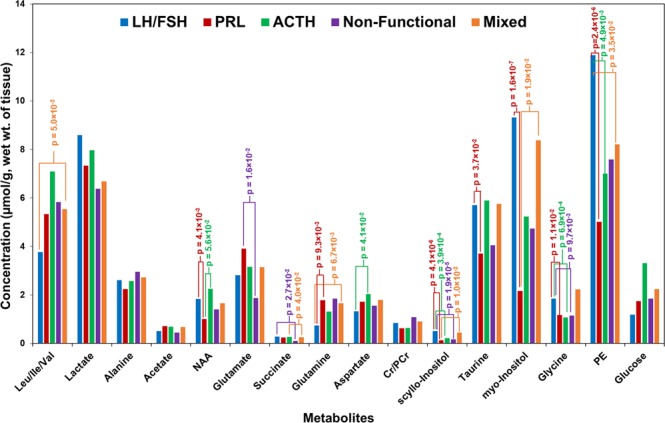
Chart showing average concentrations of aqueous metabolites (expressed in µmol/g, wet weight of tissue) detected in various pituitary tumors. Since LH and FSH secreting tumors both produce gonadotropins, they have been combined together in the quantitative analysis.

Figure 2 shows average concentrations of aqueous metabolites (µmol/g, wet weight of tissue) in LH/FSH, PRL, ACTH, non-functional and mixed-hormone (plurihormonal) PAs (more details in Supplementary Table S1). Given LH and FSH both are gonadotropins, we have combined them together for the analysis. Many metabolites showed statistically significant differences while comparing between different subtypes. p-values are given in the Table S2. Average concentrations of metabolites in LH/FSH group were compared with those in PRL, ACTH, non-functional and mixed PAs. The levels of NAA, mI, glycine, taurine and PE were decreased in PRL when compared to LH/FSH-secreting tumors. sI was found to be lower in PRL, ACTH, non-functional and mixed tumors compared to LH/FSH-secreting tumors (Tables S1 and S2).

NAA is a key metabolite present in the brain specific to central nervous system metabolism^16^. Its concentration in certain parts of the brain can reach upto 10.0 mM. The biological function of NAA is not well understood, but experimental evidences suggest that it acts as an osmolyte, removes metabolically produced water from neurons, and an acetyl group provider for lipid synthesis including other functions such as facilitator of glutamine/glutamate metabolism in neuronal mitochondria, bypassing the glutamate dehydrogenase, and altering metabolism in cancer cells^17,18^. In the current study, the concentration of NAA in various PAs range from 0.176–4.072 µmol/g, wet weight of tissue. Literature data from *in vivo* ^1^H NMR study of pituitary tumors demonstrates that NAA levels were reduced in majority of the pituitary tumors irrespective of their subtype^19^. However, our current data shows that NAA levels are lower by 45.6% in PRL, 23.8% in non-functional and 10.2% in mixed tumors when compared to LH/FSH-secreting tumors (Fig. 2 and Table S1). A recent MS study also revealed a significant decrease in the levels of NAA in pituitary tumors compared to the white and gray matter of human tissue specimens^8^. NAA is synthesized from aspartate and acetyl-CoA. Aspartate levels in different PAs are found to be similar (Fig. 2). Lower levels of NAA could also be due to active degradation or catabolism of NAA. NAA degradation by aspartoacylase (ASPA) generates acetate and aspartate. Detection of relatively higher amounts of acetate in PRLs compared to other subtypes (Fig. 2) could be due to the presence of an active NAA catabolism pathways in PRL secreting tumors. Similarly, slightly elevated levels of aspartate, NAA and acetate in ACTH secreting tumors indicate active NAA metabolism. Together, these data indicate that NAA may play a key role in energy metabolism of pituitary tumors through modulation of acetyl-CoA and aspartate pools in these tumors.

Lower levels of lactate (25.8%) and glycine (37.7%) in non-functional tumors (difference in the lactate levels is statistically non-significant) compared to LH/FSH-secreting tumors may indicate a reduced flux through glycolysis in non-functional tumors. Observation of relatively higher levels of glutamate and glutamine only in PRL secreting tumors may be due to the presence of active mitochondrial oxidation of glucose through pyruvate. Also, this may raise the possibility of the mitochondrial oxidation of other nutrients such as ketone bodies and free fatty acids in PRL secreting tumors.

The isomeric sugars mI and sI were decreased in PRL, ACTH secreting, non-functional and mixed tumors compared to LH/FSH group. The decrease in the levels of mI was statistically significant only in PRL compared to LH/FSH, whereas the decrease in sI was statistically significant in four different comparisons (LH/FSH vs. PRL, ACTH, non-functional; mixed vs PRL, Table S2). Previous *in vivo* magnetic resonance spectroscopy (MRS) studies on brain diseases have also observed a simultaneous decrease in both mI and sI^20^. Similar to LH/FSH-secreting tumors, elevated levels of mI were also reported in some gliomas and metastatic brain tumor patients^9^. mI is involved in the biosynthesis of glucuronate, phosphatidylinositol and inositol polyphosphates which have important role in cell signaling. sI may be generated from the larger pool of mI that was present in pituitary tumors. It was recently shown that sI can block amyloid formation in mouse brain^21^. Current findings that the decreased levels of mI and sI in PAs except LH/FSH subtype indicate downregulated inositol metabolism.

In a previous study, authors compared levels of PE in human normal cortex and pituitary tumors and they found that PE was in higher concentration in pituitary tumors than in the normal cortex^9^. The histopathological subtypes of pituitary tumors in their study was not described. In the current study, we have observed decreased levels of PE in PRL, ACTH secreting, non-functional and mixed tumors compared to LH/FSH group. PE is highly abundant in pituitary gland^22^, and lower levels of PE in the above subtypes indicate dysregulated metabolism of PE in these tumor subtypes. In another study, a human neoplastic mammary epithelial cell line (T47-D) gave a positive growth response to PE. Moreover, this response of mammary epithelial cells to PE was correlated with the growth response of this cell line to the presence of PRL in culture^23^. In the current study, the decrease in the levels of PE may be due to the elevated levels of PRL in PRL secreting tumors (Fig. 2).

### Lipid components in PAs

**Figure 3:**
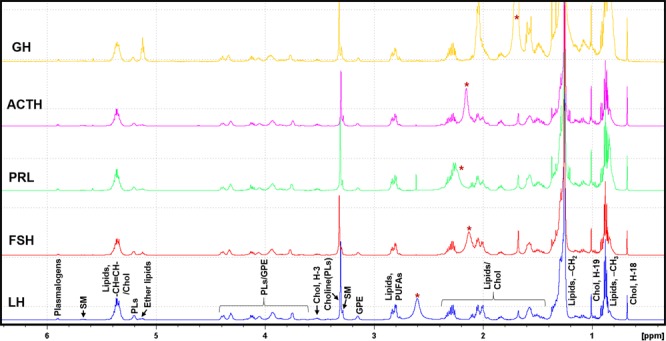
Typical ^1^H NMR spectra of chloroform layer of methanol:chloroform (2:1, v/v) extracts of LH, FSH, PRL, ACTH and GH secreting pituitary tumors showing relative levels of various lipid components. Chol, cholesterol; PUFAs, polyunsaturated fatty acids; GPE, glycerophosphoethanolamine; PLs, phospholipids; choline(PLs), choline containing phospholipids; SM, sphingomyelin, GPE, glycerophosphoethanolamine; (*: solvent impurity).

**Figure 4:**
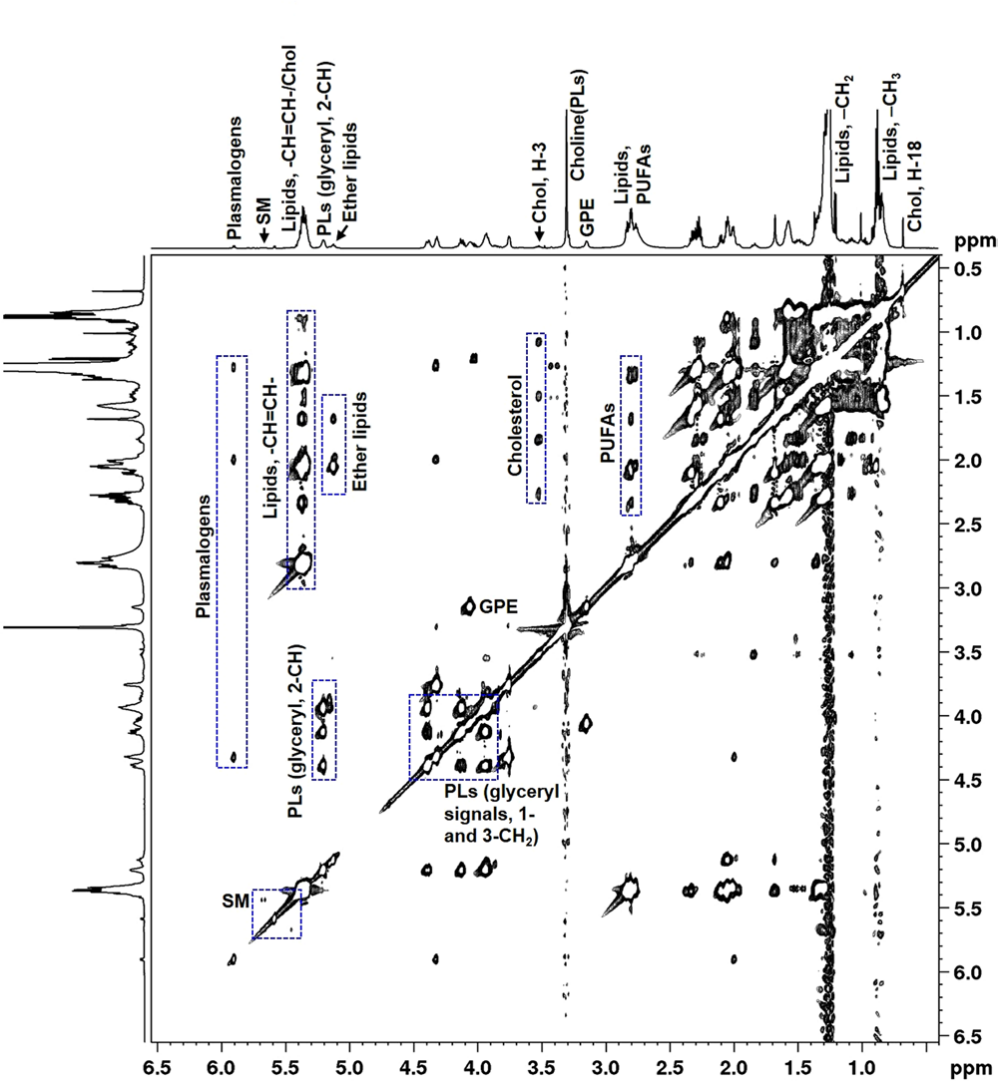
A typical 2D ^1^H-^1^H total correlated spectroscopy (TOCSY) spectra of chloroform layer of methanol:chloroform (2:1, v/v) extract of PA showing unambiguous identification various lipid components.

Figure 3 illustrates ^1^H NMR spectra of CHCl_3_ extracts of tumor tissues showing the presence of the following membrane lipids: cholesterol, polyunsaturated fatty acids (PUFAs), glycerophosphoethanolamine (GPE), choline containing phospholipids [choline(PLs)], sphingomyelin (SM), phospholipids (PLs), ether lipids (plasmanyl lipids and plasmalogens). These classes of lipids were identified by a detailed spectroscopic analysis of 1D ^1^H and 2D ^1^H-^1^H TOCSY NMR experiments and assignments were subsequently compared with the reported literature^24,25,^. Figure 4 shows 2D ^1^H-^1^H TOCSY spectrum of a representative chloroform extract with peak assignments of various lipid molecules.

**Figure 5:**
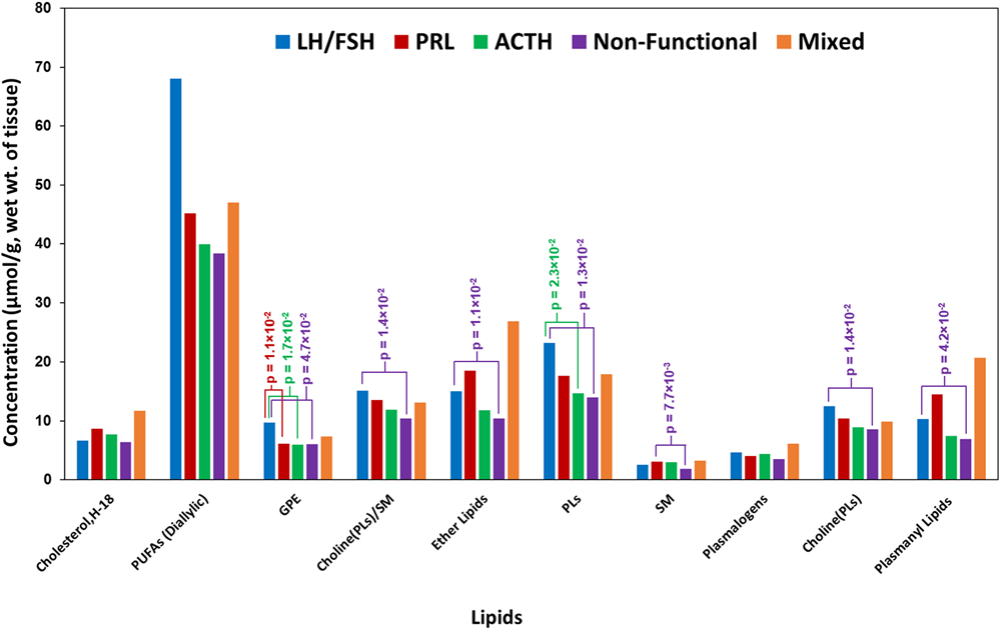
Chart showing average concentrations of lipid components (expressed in µmol/g, wet weight of tissue) detected in various pituitary tumors.

Figure 5 shows average concentrations of lipid components identified in various subtypes of PAs (Table S3). The following lipid molecules showed statistically significant differences when compared between different subtypes and the corresponding p-values are given in Table S4: **1**. GPE was lower in PRL, ACTH, non-functional and mixed subtypes when compared to LH/FSH. **2**. Choline(PLs) and plasmanyl lipids were lower in non-functional tumors compared to LH/FSH-secreting tumor type (Fig. 5). **3.** In ACTH-secreting tumor, PLs were lower in all the subtypes when compared to LH/FSH-secreting tumors. We have observed that the ether lipids (specifically, plasmanyl-GPC/GPE) resonating at 5.11 ppm along with plasmalogens (plasmenyl-GPC/GPE) have elevated in PRL secreting and mixed tumors (Fig. 5) compared to the rest of the subtypes. Ether lipids are an important class of glycerophospholipids present in cell membranes which play a major role in the cholesterol biosynthesis^26^. In this study, we have observed elevated levels of both ether lipids and cholesterol in PRL-secreting and mixed tumors. Monitoring the levels of these classes of lipids in large patient cohort would help to unravel a correlation between altered levels of ether lipids on cholesterol homeostasis.

## Conclusion

Current NMR studies on various immunohistochemical subtypes of PAs revealed statistically significant decrease in the levels of NAA, mI, sI, glycine, taurine, PE and increase in the levels of glutamine in PRL secreting tumors when compared with LH/FSH-secreting tumors. On the other hand, statistically significant decrease in the levels of sI, glycine, PE and increase in the levels of aspartate were observed between ACTH and LH/FSH-secreting tumors. Although lipid extracts of PAs showed the presence of many common lipid molecules, only GPE showed statistically significant decrease in PRL, ACTH and non-functional subtypes when compared to LH/FSH-secreting tumors. These observed differences can be attributed to the changes in flux through various metabolic pathways involving these metabolites in pituitary tumors. Also, our *ex vivo* observation of detecting elevated levels of the peptide ACTH in pituitary tumor patients with Cushing’s disease, can be translated to *in vivo* ^1^H-MRS using MRI scanners for the non-invasive diagnosis of Cushing’s disease. Further work is warranted with increased sample size in particular from ACTH-secreting and non-functional tumors. We are continuing the recruitment of patients from all histological subtypes of pituitary tumors to further validate our findings from the current study.

## Supplementary information

Supplementary data

## Data Availability

All data generated or analyzed during this study are given in this article and its supplementary information file.
