# Supplementary material for: Ex Vivo1H NMR study of pituitary adenomas to differentiate various immunohistochemical subtypes
Source: Sci Rep. 2019 Feb 28;9:3007. doi: 10.1038/s41598-019-38542-6 (PMC6395808; doi:10.1038/s41598-019-38542-6)
Supplement: Supplementary file 1 — Supplementary data [file 41598_2019_38542_MOESM1_ESM.docx]

***Ex Vivo* ^1^H NMR study of pituitary adenomas to differentiate various immunohistochemical subtypes**

Omkar B. Ijare^1^, David S. Baskin^1,2,*^, and Kumar Pichumani^1,2,*^

^1^Kenneth R. Peak Brain and Pituitary Tumor Treatment Center, Department of Neurosurgery, Houston Methodist Neurological Institute, Houston Methodist Hospital and Research Institute, Houston, TX, USA, ^2^Weill Cornell Medical College, New York, NY, USA.

| **Metabolite (µmol/g wet wt. of tissue),**  Mean ± S.D. | **^1^H NMR Chemical Shift (ppm)** | **FSH, LH,**  **LH/FSH**  **(n=17)** | **PRL**  **(n=11)** | **ACTH**  **(n=4)** | **Non-functional**  **(n=5)** | **Mixed**  **(n=8)** |
| --- | --- | --- | --- | --- | --- | --- |
| Leu/Ile/Val | 0.851 | 3.773 ± 1.342 | 5.334 ± 2.534 | 7.088 ± 3.729 | 5.827 ± 4.425 | 5.539 ± 2.353 |
| Lactate | 1.317 | 8.601 ± 4.004 | 7.333 ± 4.425 | 7.972 ± 3.436 | 6.378 ± 3.099 | 6.679 ± 2.430 |
| Alanine | 1.469 | 2.614 ± 1.153 | 2.246 ± 1.194 | 2.574 ± 0.746 | 2.960 ± 1.905 | 2.726 ± 1.296 |
| Acetate | 1.905 | 0.510 ± 0.262 | 0.714 ± 0.665 | 0.690 ± 0.210 | 0.442 ± 0.235 | 0.674 ± 0.470 |
| NAA | 2.007 | 1.841 ± 0.926 | 1.002 ± 0.467 | 2.261 ± 0.868 | 1.403 ± 1.396 | 1.654 ± 0.769 |
| Glutamate | 2.344 | 2.816 ± 1.113 | 3.916 ± 1.662 | 3.165 ± 1.511 | 1.872 ± 1.149 | 3.143 ± 1.361 |
| Succinate | 2.394 | 0.281 ± 0.127 | 0.243 ± 0.133 | 0.264 ± 0.051 | 0.100 ± 0.125 | 0.252 ± 0.067 |
| Glutamine | 2.439 | 0.744 ± 0.657 | 1.789 ± 1.037 | 1.311 ± 1.023 | 1.849 ± 1.275 | 1.663 ± 0.784 |
| Aspartate | 2.806 | 1.321 ± 0.800 | 1.716 ± 1.030 | 2.044 ± 0.455 | 1.554 ± 1.306 | 1.798 ± 0.868 |
| Cr/PCr | 3.031 | 0.839 ± 0.444 | 0.619 ± 0.304 | 0.640 ± 0.074 | 1.085 ± 1.308 | 0.912 ± 0.442 |
| scyllo-Inositol | 3.335 | 0.510 ± 0.237 | 0.126 ± 0.053 | 0.212 ± 0.071 | 0.161 ± 0.054 | 0.449 ± 0.264 |
| Taurine | 3.404 | 5.702 ± 1.933 | 3.706 ± 2.488 | 5.893 ± 3.299 | 4.048 ± 2.151 | 5.754 ± 3.796 |
| myo-Inositol | 3.521 | 9.323 ± 3.505 | 2.163 ± 0.867 | 5.238 ± 2.980 | 4.733 ± 5.230 | 8.383 ± 5.770 |
| Glycine | 3.546 | 1.847 ± 0.755 | 1.173 ± 0.550 | 1.075 ± 0.077 | 1.151 ± 0.336 | 2.235 ± 1.152 |
| PE | 3.967 | 11.891 ± 3.729 | 5.018 ± 2.327 | 7.000 ± 1.913 | 7.591 ± 6.068 | 8.207 ± 4.234 |
| α-Glucose | 5.215 | 0.428 ± 0.387 | 0.629 ± 0.394 | 1.192 ± 1.055 | 0.665 ± 0.382 | 0.807 ± 0.554 |
| Glucose* |  | 1.189 ± 1.074 | 1.747 ± 1.096 | 3.312 ± 2.930 | 1.847 ± 1.062 | 2.241 ± 1.540 |

**Table S1**: Concentrations of metabolites (µmol/g, wet wt. of tissue) in the aqueous methanol layers of methanol:chloroform (2:1, v/v) extracts of pituitary adenomas.

*Glucose concentration was determined from α-Glucose (36% anomeric contribution) using the relation, [Glucose] = [α-Glucose]×(100/36).

**Table S2**: P-values of comparisons of metabolite concentrations (from Table S1) in various pituitary tumor subtypes. A p-value ≥0.05 (5.0E-02) was considered statistically significant.

| **Metabolite** | **(FSH,LH,**  **LH/FSH) vs. PRL** | **(FSH,LH,**  **LH/FSH) vs. ACTH** | **(FSH,LH,**  **LH/FSH) vs. Non-Functional** | **FSH, LH, LH/FSH vs. Mixed** | **PRL vs. ACTH** | **PRL vs. Non-Functional** | **PRL vs. Mixed** | **ACTH vs. Non-Functional** | **ACTH vs. Mixed** | **Mixed vs. Non-Functional** |
| --- | --- | --- | --- | --- | --- | --- | --- | --- | --- | --- |
| Leu/Ile/Val | 8.2E-02 | 1.7E-01 | 3.6E-01 | 5.0E-02 | 4.3E-01 | 8.3E-01 | 8.6E-01 | 6.2E-01 | 4.9E-01 | 9.0E-01 |
| Lactate | 4.5E-01 | 7.6E-01 | 2.2E-01 | 1.3E-01 | 7.8E-01 | 6.3E-01 | 6.9E-01 | 4.3E-01 | 5.3E-01 | 8.6E-01 |
| Alanine | 4.3E-01 | 9.3E-01 | 7.2E-01 | 8.2E-01 | 5.4E-01 | 4.7E-01 | 4.2E-01 | 6.9E-01 | 8.0E-01 | 8.2E-01 |
| Acetate | 3.5E-01 | 2.0E-01 | 5.9E-01 | 3.3E-01 | 9.2E-01 | 2.5E-01 | 8.8E-01 | 1.0E-01 | 9.4E-01 | 2.6E-01 |
| NAA | 4.1E-03 | 4.3E-01 | 5.4E-01 | 5.8E-01 | 5.6E-02 | 5.6E-01 | 5.7E-02 | 2.7E-01 | 2.9E-01 | 7.3E-01 |
| Glutamate | 7.1E-02 | 6.9E-01 | 1.5E-01 | 5.3E-01 | 4.4E-01 | 1.6E-02 | 2.8E-01 | 1.4E-01 | 9.8E-01 | 1.0E-01 |
| Succinate | 4.7E-01 | 6.9E-01 | 2.7E-02 | 4.5E-01 | 6.6E-01 | 7.0E-02 | 8.5E-01 | 4.0E-02 | 7.4E-01 | 5.0E-02 |
| Glutamine | 9.3E-03 | 3.6E-01 | 1.3E-01 | 6.7E-03 | 4.6E-01 | 9.3E-01 | 7.7E-01 | 4.6E-01 | 5.7E-01 | 7.8E-01 |
| Aspartate | 3.0E-01 | 4.1E-02 | 7.2E-01 | 1.7E-01 | 4.1E-01 | 8.2E-01 | 8.5E-01 | 4.6E-01 | 5.3E-01 | 7.2E-01 |
| Cr/PCr | 1.3E-01 | 9.6E-02 | 7.0E-01 | 6.8E-01 | 8.4E-01 | 4.7E-01 | 1.3E-01 | 4.9E-01 | 1.3E-01 | 7.9E-01 |
| scyllo-Inositol | 4.1E-06 | 3.9E-04 | 1.9E-05 | 5.5E-01 | 8.7E-02 | 2.5E-01 | 1.0E-02 | 2.0E-01 | 4.3E-02 | 1.8E-02 |
| Taurine | 3.7E-02 | 9.2E-01 | 1.7E-01 | 9.7E-01 | 2.9E-01 | 7.9E-01 | 2.1E-01 | 2.8E-01 | 9.5E-01 | 3.2E-01 |
| myo-Inositol | 1.6E-07 | 6.1E-02 | 1.2E-01 | 6.5E-01 | 1.3E-01 | 3.3E-01 | 1.9E-02 | 8.5E-01 | 2.4E-01 | 2.7E-01 |
| Glycine | 1.1E-02 | 6.9E-04 | 9.7E-03 | 3.5E-01 | 5.8E-01 | 9.2E-01 | 3.8E-02 | 6.4E-01 | 2.5E-02 | 3.5E-02 |
| PE | 2.4E-06 | 4.5E-03 | 1.9E-01 | 3.6E-02 | 1.4E-01 | 4.0E-01 | 8.2E-02 | 8.4E-01 | 5.1E-01 | 8.5E-01 |
| alpha-Glucose | 2.0E-01 | 2.4E-01 | 2.7E-01 | 7.7E-02 | 3.7E-01 | 8.7E-01 | 4.5E-01 | 2.8E-01 | 5.3E-01 | 6.0E-01 |
| Glucose | 2.0E-01 | 2.4E-01 | 2.7E-01 | 7.7E-02 | 3.7E-01 | 8.7E-01 | 4.5E-01 | 2.8E-01 | 5.3E-01 | 6.0E-01 |

**Table S3**: Concentrations of lipid components (µmol/g, wet wt. of tissue) in the chloroform layers of methanol:chloroform (2:1, v/v) extracts of pituitary adenoma.

| **Lipids (µmol/g, wet wt. of tissue),**  **Mean ± S.D.** | **^1^H NMR Chemical shift (ppm)** | **LH,FSH,LH/FSH**  **(n=16)** | **PRL**  **(n=11)** | **ACTH**  **(n=4)** | **Non-functional**  **(n=5)** | **Mixed**  **(n=8)** |
| --- | --- | --- | --- | --- | --- | --- |
| Cholesterol, H-18 | 0.676 | 6.636 ± 2.260 | 8.704 ± 4.175 | 7.732 ± 2.810 | 6.403 ± 2.186 | 11.707 ± 10.818 |
| PUFAs (Diallylic) | 2.758 | 68.047 ± 67.166 | 45.174 ± 17.421 | 39.933 ± 10.719 | 38.399 ± 11.040 | 47.058 ± 18.463 |
| GPE | 3.148 | 9.720 ± 4.597 | 6.178 ± 1.821 | 6.014 ± 1.550 | 6.040 ± 2.684 | 7.384 ± 2.009 |
| Choline(PLs) + SM | 3.311 | 15.122 ± 5.590 | 13.534 ± 4.779 | 11.945 ± 3.353 | 10.469 ± 2.153 | 13.171 ± 4.862 |
| Ether Lipids | 5.107 | 15.038 ± 6.291 | 18.549 ± 12.246 | 11.824 ± 3.824 | 10.451 ± 0.576 | 26.868 ± 23.720 |
| PLs (glyceryl 2-CH) | 5.217 | 23.243 ± 9.980 | 17.717 ± 6.068 | 14.696 ± 4.266 | 14.046 ± 4.710 | 17.956 ± 5.302 |
| SM | 5.675 | 2.578 ± 1.202 | 3.115 0.971 | 3.007 ± 1.308 | 1.888 ± 0.569 | 3.293 ± 2.399 |
| Plasmalogens | 5.900 | 4.653 ± 1.614 | 4.041 ± 1.485 | 4.366 ± 1.716 | 3.509 ± 1.138 | 6.161 ± 3.649 |
| ^#^Choline(PLs) |  | 12.544 ± 4.607 | 10.419 ± 4.173 | 8.938 ± 2.662 | 8.582 ± 1.919 | 9.878 ± 2.521 |
| ^##^Plasmanyl Lipids |  | 10.384 ± 5.913 | 14.508 ± 12.381 | 7.458 ± 5.239 | 6.942 ± 1.277 | 20.706 ± 21.619 |

^#^Choline(PLs) = [Choline(PLs) + SM] – SM; ^##^Plasmanyl lipids = Ether lipids – Plasmalogens; PLs. Phospholipids;

**Table S4**: P-values of comparisons of lipid concentrations (from Table S3) in various pituitary tumor subtypes. A p-value ≥0.05 (5.0E-02) was considered statistically significant.

| **Lipids (µmol/g, wet wt. of tissue)** | (FSH,LH,  LH/FS)  vs. PRL | (FSH,LH,  LH/FSH)  vs. ACTH | FSH,LH,  LH/FSH)  vs. Non-Functional | FSH, LH,  LH/FSH)  vs. Mixed | PRL vs.  ACTH | PRL vs.  Non-Functional | PRL vs. Mixed | ACTH vs. Non-Functional | ACTH vs. Mixed | Mixed vs.  Non-Functional |
| --- | --- | --- | --- | --- | --- | --- | --- | --- | --- | --- |
| Cholesterol, H-18 | 1.6E-01 | 5.1E-01 | 8.4E-01 | 2.3E-01 | 6.2E-01 | 1.7E-01 | 4.8E-01 | 4.7E-01 | 3.6E-01 | 2.2E-01 |
| PUFAs (Diallylic) | 3.7E-01 | 1.4E-01 | 8.2E-02 | 5.6E-01 | 5.0E-01 | 3.7E-01 | 8.3E-01 | 8.4E-01 | 4.2E-01 | 3.1E-01 |
| GPE | 1.1E-02 | 1.7E-02 | 4.7E-02 | 9.8E-02 | 8.7E-01 | 9.2E-01 | 2.0E-01 | 9.9E-01 | 2.3E-01 | 3.7E-01 |
| Choline(PLs) + SM | 4.4E-01 | 1.8E-01 | 1.4E-02 | 3.9E-01 | 4.9E-01 | 9.9E-02 | 8.7E-01 | 4.8E-01 | 6.2E-01 | 2.0E-01 |
| Ether Lipids | 4.0E-01 | 2.3E-01 | 1.1E-02 | 2.1E-01 | 1.3E-01 | 5.3E-02 | 3.9E-01 | 5.3E-01 | 1.2E-01 | 9.1E-02 |
| PLs (glyceryl 2-CH) | 8.6E-02 | 2.3E-02 | 1.3E-02 | 1.0E-01 | 3.1E-01 | 2.2E-01 | 9.3E-01 | 8.3E-01 | 2.9E-01 | 2.0E-01 |
| SM | 2.1E-01 | 5.8E-01 | 1.0E-01 | 4.5E-01 | 8.9E-01 | 7.7E-03 | 8.5E-01 | 1.9E-01 | 8.0E-01 | 1.5E-01 |
| Plasmalogens | 3.2E-01 | 7.8E-01 | 1.1E-01 | 3.0E-01 | 7.5E-01 | 4.5E-01 | 1.6E-01 | 4.3E-01 | 2.7E-01 | 8.8E-02 |
| Choline(PLs) | 2.3E-01 | 7.4E-02 | 1.4E-02 | 8.1E-02 | 4.4E-01 | 2.5E-01 | 7.3E-01 | 8.3E-01 | 5.8E-01 | 3.2E-01 |
| Plasmanyl lipids | 3.2E-01 | 3.7E-01 | 4.3E-02 | 2.2E-01 | 1.5E-01 | 7.2E-02 | 4.8E-01 | 8.6E-01 | 1.4E-01 | 1.2E-01 |


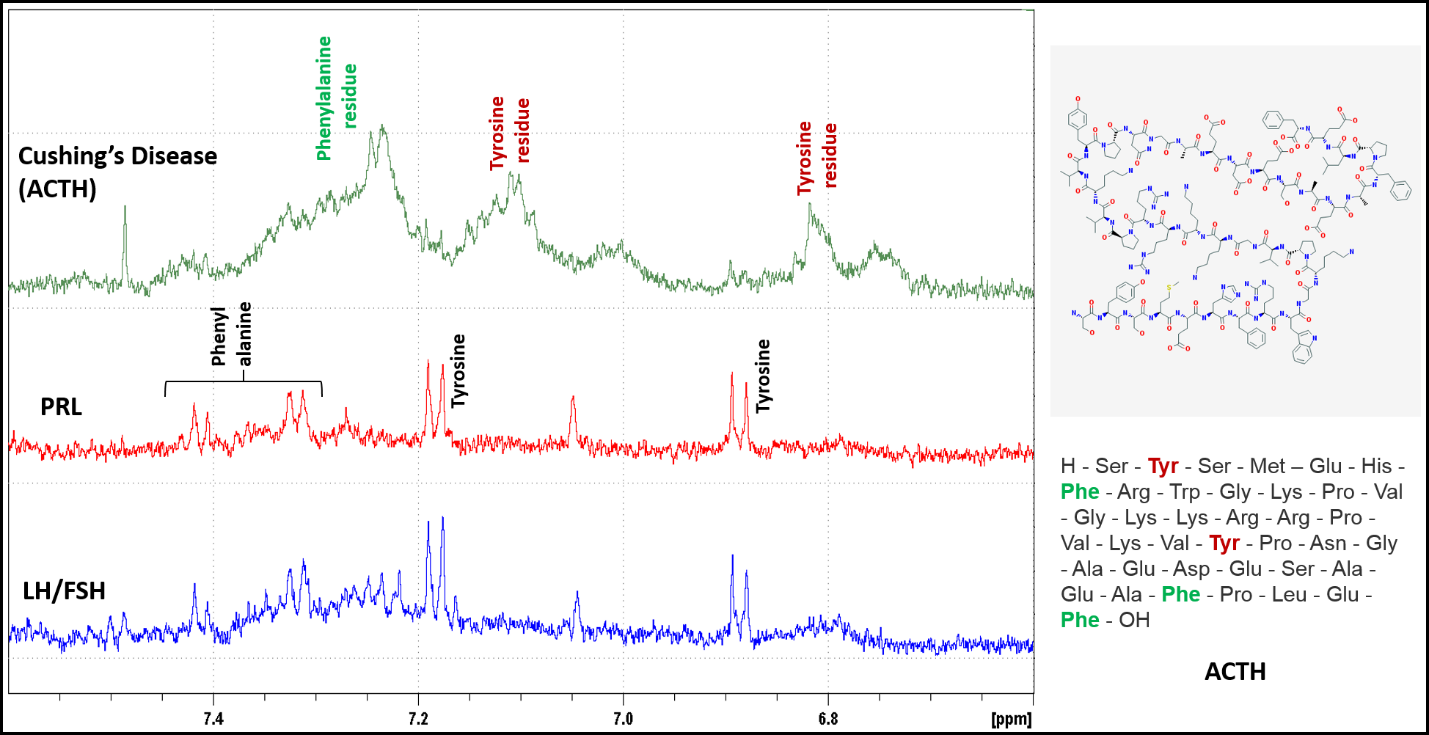


**Figure S1:** Representative ^1^H NMR spectra (aromatic region, 6.70 – 7.40 ppm) of methanol:chloroform (2:1) extracts from LH/FSH, PRL, and ACTH secreting tumors. ACTH-secreting tumors with Cushing’s disease showed the presence of elevated levels of phenylalanine and tyrosine residues, arising from ACTH hormone, as broad signals. These Cushing’s disease-specific ^1^H-NMR signatures can be used in differential diagnosis of ACTH from other pituitary tumors.
